# Influence of the Interaction between Genetic Factors and Breastfeeding on Children’s Weight Status: A Systematic Review

**DOI:** 10.1016/j.advnut.2024.100312

**Published:** 2024-10-09

**Authors:** Zhe Yang, Na Li, Hong Cui, Borui Liu, Xue Wang, Ling Zhang, Xiaochuan Wang, Lu Zheng, Xinyue Yang, Shuqi Wu, Jiajin Hu, Deliang Wen

**Affiliations:** 1 Health Sciences Institute, China Medical University, Shenyang, China; 2 Research Center of China Medical University Birth Cohort, China Medical University, Shenyang, China; 3 Liaoning Key Laboratory of Obesity and Glucose/Lipid Associated Metabolic Diseases, China Medical University, Shenyang, China; 4 Department of Obstetrics and Gynecology, Shengjing Hospital of China Medical University, China Medical University, Shenyang, China; 5 Medical Information Research Department/Library, Xuanwu Hospital, Capital Medical University, Beijing, China; 6 Department of Epidemiology and Health Statistics, School of Public Health, Capital Medical University, Beijing, China

**Keywords:** breastfeeding, children, obesity, heredity, interaction, weight status

## Abstract

Breastfeeding may interact with other risk factors and have a combined influence on child growth. This systematic review aimed to examine the interaction between genetic factors and breastfeeding and how their combination is associated with children weight status. Four databases were searched until August 2024, and 8 eligible studies were identified. The fat mass and obesity associated (*FTO*) and peroxisome proliferator-activated receptor γ2 (*PPARG2*) genes were the most examined genes. Although the results of interactions between breastfeeding and genetics factors on children’s weight status were inconsistent, some of studies reported that breastfeeding or exclusive breastfeeding attenuated the disadvantageous association between the risk alleles of the genes (higher obesity-specific genetic risk score for a multiple-gene study) and overdevelopment of children’s body weight. These findings support the WHO recommendations for prolonged breastfeeding and further suggest breastfeeding interventions to prevent childhood obesity may be more effective in populations with a disadvantageous genetic predisposition.

This review was registered in PROSPERO as CRD42023448365.

**Statement of signiﬁcance**

To our knowledge, this is the first systematic review to critically evaluate the interaction between breastfeeding and genetic factors and their influence on weight status in children.

## Introduction

Obesity is a major public health challenge worldwide. Childhood obesity not only affects the physical development of children, but it can also lead to cardiovascular diseases or metabolic-related diseases in adulthood [1]. Obesity is a chronic disease that is influenced by both genetic and environmental factors [2]. As a modifiable behavioral factor in early life, breastfeeding for ≥6 mo is recommended by the WHO and the International Children’s Fund because of its beneficial health effects on child development, which include the prevention of childhood obesity [3].

Breastfeeding can modify gene expression levels, thus having an impact on the development of childhood obesity [4]. For example, breast milk has been reported to alter the DNA methylation of the fat mass and obesity associated (*FTO*) gene, which is the gene most strongly associated with obesity because it has by far the largest effect size and has the largest explained variance among individuals of European ancestry [5]. The product of the *FTO* gene regulates energy metabolism by inhibiting m6A demethylase activity, which suggests that breastfeeding may alter *FTO* gene expression through the transfer of milk-derived exosomes [6], thus altering the expression of genes and the development of obesity through epigenetic modification. However, existing epidemiological studies examining the interaction between breastfeeding and obesity-related genes have reported inconsistent results. For example, some studies have reported that breastfeeding reduces the risk of obesity in children carrying mutations in obesity-related genes [4,7]; however, another study did not observe a significant relationship between breastfeeding and the risk of obesity [8]. These conflicting findings underscore the interplay between breastfeeding and obesity-related genes. One possible explanation for the discrepancy is the focus on different genes across the studies, considering that obesity is a polygenic disease [9,10]. Furthermore, the expression of these genes may vary depending on sensitivity to breastfeeding.

In addition, the interaction association between breastfeeding and gene expression may be dependent on breastfeeding duration [11,12], which can range from rarely to >24 mo [8]. Therefore, there may be an exposure–response association between breastfeeding duration and gene expression; a longer breastfeeding time could alter the role of genes in the development of obesity.

To our knowledge, no previous study has systematically reviewed the interaction between breastfeeding and genotypes and their influence on the development of childhood obesity. This is important because it could help identify populations with a specific genetic background that are sensitive to breastfeeding. Furthermore, the findings can be used to develop a target intervention strategy to prevent childhood obesity in early life. In this systematic review, we aimed to demonstrate the interaction between genotypes and breastfeeding and how their combination influences weight status and obesity development in children.

## Methods

This systematic review was conducted according to PRISMA 2020, including the procedures for the identification, screening, and determination of eligibility of studies [13]. The review protocol was registered at PROSPERO (registration number: CRD42023448365).

### Eligibility criteria

This systematic review included cohort, cross-sectional, and case-control studies. In the present study, we defined breastfeeding–gene interaction as the nonindependent association of the 2 exposures on children’s weight status, and we aimed to examine whether the association between one exposure factor (e.g., a gene) and children’s weight status differs depending on the level of the other exposure factor (e.g., breastfeeding). The gene–breastfeeding interaction association was determined or measured by using the interaction term of the 2 exposures across all the studies, and the significance of the interaction association was determined according to the *P* value of the interaction term, at the significance level of 0.05. Based on the PECO (population, exposure, comparison, outcome) model [13], the inclusion criteria were as follows: *1*) population: child aged 0–20 y; *2*) exposure: exclusive breastfeeding or any breastfeeding and the genetics of the child; and *3*) outcome: child weight status. The exclusion criteria were as follows: *1*) studies that did not report the interaction between genes and breastfeeding on weight status in children; *2*) duplicate publications; *3*) animal studies, case reports, conference abstracts, or reviews; *4*) low-quality studies; and *5*) inability to access the full text or complete statistical data despite efforts to contact the original authors.

### Search strategy

Extensive searches were conducted in PubMed, Embase, Cochrane, and Web of Science electronic databases for articles published before August 2024. The search was limited to English-language studies published in peer-reviewed journals. The entire search strategy is presented in Supplementary Tables 1 and 4. We also conducted reverse snowballing [14] (using a list of references that incorporate an article to identify additional articles) on review articles retrieved with our search strategy.

### Study selection

**FIGURE 1:**
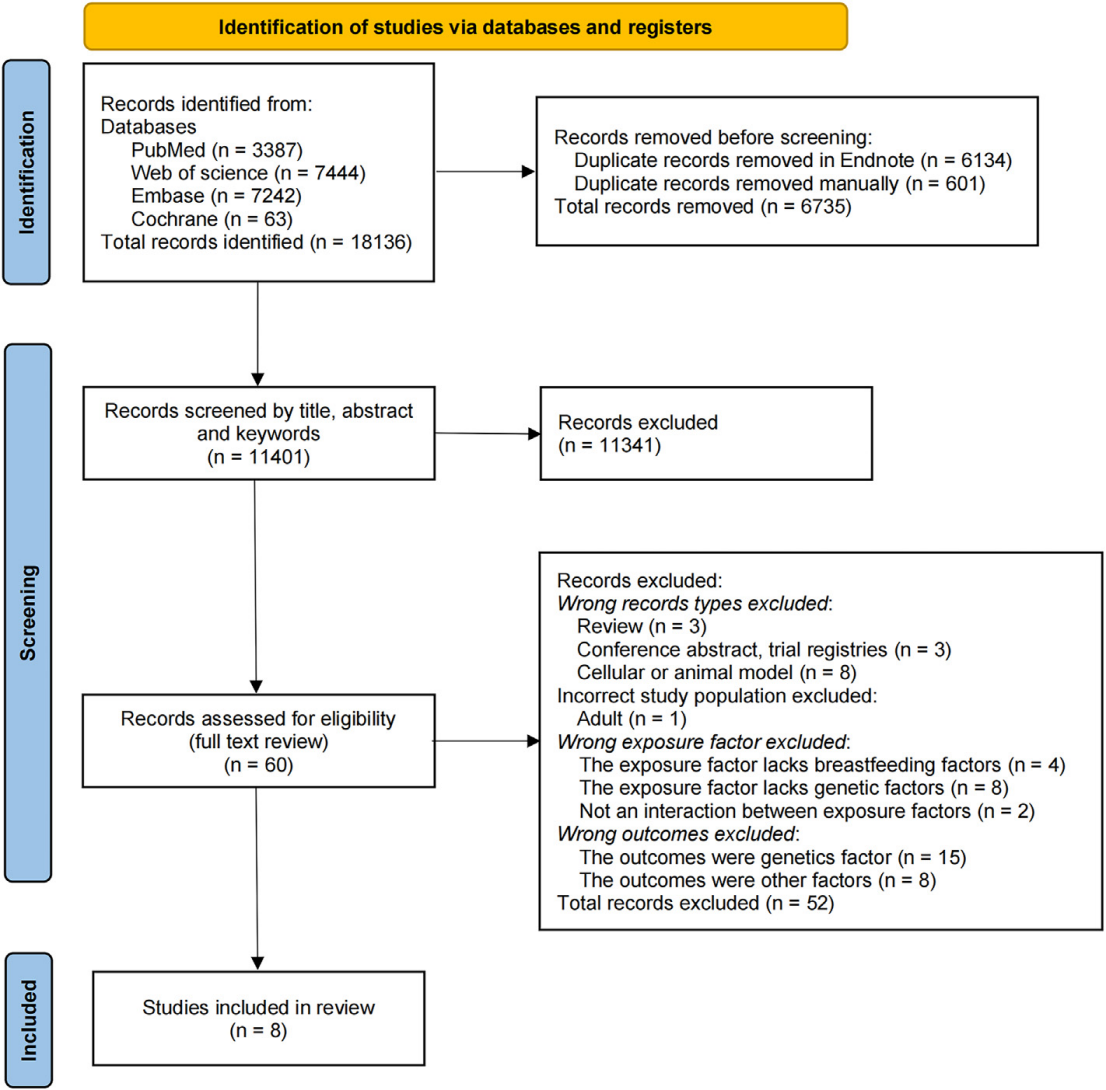
Flowchart of the search: PRISMA diagram.

As per the protocol, we conducted a methodical search of the above databases and exported the results to EndNote (X9). Duplicate records were systematically reviewed and removed using the Bramer method [15]. The titles and abstracts were independently screened by 2 reviewers, and irrelevant studies were removed based on the eligibility criteria. The decisions made by the 2 reviewers were checked, and Cohen’s κ statistics were generated [16]. The researchers were in general agreement (κ: 0.842; 95% confidence interval: 0.766, 0.919; *P* < 0.001), with a 99.86% agreement rate. The full text of the resulting literature was thoroughly reviewed by 2 reviewers who independently selected the studies and reached a consensus with a third reviewer to resolve differences through discussion. Decisions regarding the final studies that were included were made by all reviewers. The reasons for exclusion are shown in Figure 1.

### Data extraction

Data extracted from the included studies included publication information, study design, study participant characteristics, exposure, outcomes, and findings. The preliminary results were extracted independently by one reviewer and were discussed by the research team to ensure an error-free data extraction process and consistent study results. The results were imported into an Excel spreadsheet for data integration.

### Quality assessment

Study quality was evaluated using modified versions of the Newcastle–Ottawa Scale (NOS) [17] and the Agency for Healthcare Research and Quality (AHRQ) [18] assessment tools. The NOS scoring criteria were used to assess the quality of the cohort studies, which consisted of a maximum of 9 points per study: 4 for selection, 2 for comparability, and 3 for outcome assessment. Article quality was assessed as follows: total scores of 0 to 3, 4 to 6, and 7 to 9 represented low-, medium-, and high-quality studies, respectively. The quality of the cross-sectional studies was assessed using the AHRQ scoring criteria, which comprises an 11-item checklist. Article quality was assessed as follows: total scores of 0 to 3, 4 to 7, and 8 to 11 represented low-, medium-, and high-quality studies, respectively. In the present study, all selected studies achieved high- or medium-quality and were included in the final analysis (Supplementary Tables 5 and 6). To ensure the quality of the assessment, 2 reviewers completed a quality assessment of the 8 included studies, and disagreements were resolved by consensus with a third reviewer. Cohen’s κ statistics could not be calculated because of the small sample size; however, the assessments of all 3 researchers were consistent across studies, and all included studies were deemed to be of high- or medium-quality.

## Results

In total, 18,136 studies were identified in the 4 databases, and 6735 were retrieved after the removal of duplicates. Sixty studies underwent review of the full text, and 8 studies were included. A flowchart of the search process is shown in Figure 1.

### Study characteristics

The year of publication of the 8 studies included in the systematic review ranged from 2009 to 2022 [[19], [20], [21], [22], [23], [24], [25], [26]]. The populations of 6 studies were European [[19], [20], [21], [22], [23], [24]], which were conducted in the United Kingdom [20,23,24], Sweden [19], Greece [20], Netherlands [22], and in 10 European cities [21]. The other studies were separately conducted in Australia [25] and China [26]. Of the 8 included studies, 6 were cohort studies [19,20,[22], [23], [24], [25]], with follow-ups at multiple time points from birth through adolescence, although the remaining 2 were cross-sectional studies [21,26]. The study characteristics are summarized in Table 1 [[19], [20], [21], [22], [23], [24], [25], [26]].

**TABLE 1:** Characteristics of included studies (*N* = 8).

Author, year, reference	Country	Design	*N*	Children’s age	Females	Genetic locus	BF categorization	Covariates	Outcome
Kanders et al., 2022 [19]	Sweden	Cohort	Wave 1: 1221; Wave 2: 1120; Wave 3: 771	Wave 1: 14.4 y; Wave 2: 17.3 y; Wave 3: 20.4 y;	Wave 1: 57.5%; Wave 2: 58.6%; Wave 3: 63.7%	*FTO* rs9939609: TT (Ref.); AT; AA	BF duration (mo): 0–6 (Ref.), 7–12, >12	Child age, child sex, and physical activity	Overweight/obesity and BMI
Dedoussis et al., 2011 [20]	GENDAI, Greece GENESIS, Greece ALSPAC, UK	Cohort	GENDAI, *n* = 1138; GENESIS, *n* = 2374; ALSPAC, *n* = 6131	GENDAI, 11.2 y; GENESIS, 3.45 y; ALSPAC, 11.7 y	GENDAI, 53.0% GENESIS,48.7% ALSPAC, 48.5%	GENDAI: *FTO* rs9939609 TT (Ref.), AT, AA GENESIS: *FTO* rs17817449 TT (Ref.), GT, GG ALSPAC: *FTO* rs9939609 TT (Ref.), AT, AA	No BF (Ref.) vs. BF	Child age, sex, Tanner stage, and physical activity	BMI, waist circumference, WHR, triceps skinfold and subscapular skinfold
Verier et al., 2010 [21]	10 European cities	Cross-sectional	945	14.7 y	54.1%	*PPARG2*: Pro12Pro (Ref.); Ala12 allele carriers	BF duration (mo): 0 (Ref.), <3, 3–5, ≥6	Age, sex, study center	BMI, waist circumference, and skinfold thicknesses
Mook-Kanamori et al., 2009 [22]	Netherlands	Cohort	3432	at birth and ages 1.5, 6, 11, 14, and 18 mo	49.2%	*PPARG2:* Pro12Pro (Ref.), Pro12Ala/ Ala12Ala	BF duration (mo): <2, 2–4, ≥4	Maternal age, educational level, parity, and weight before pregnancy	Body weight growth rate
Wu et al., 2020 [23]	UK	Cohort	5266	From birth to 17–18 y	48.9%	GRS (consisting of 94 SNPs): a range of 0–10, in which a 1-unit (i.e., 1-decile) increase in the GRS corresponded to a 4.6-allele effect in boys and 5.2 allele effect in girls	Primary analysis: EBF duration (mo): 0 (Ref.), 3, 5 Secondary analysis: BF duration (mo): 0 (Ref.), 3, 5	Gestational age, maternal preconception BMI, education, smoking status, and family income	BMI, BMI trajectory, age at adiposity peak and age at adiposity rebound
Wu et al., 2017 [24]	UK	Cohort	5590	0–16 y	49.0%	*FTO* rs9939609: TT (Ref.); AT; AA	EBF duration (mo): 0 (Ref.), ≥5	Gestational age, maternal preconception BMI, social education, and smoking status	BMI, age at adiposity peak and age at adiposity rebound
Abarin et al., 2012 [25]	Australia	Cohort	806	0–14 y	48.3%	*FTO* rs9939609: TT (Ref.), AT, AA	EBF duration (mo): 0 (Ref.), 3, 6	Gestational age, maternal preconception BMI, and mother’s education	BMI and BMI trajectory
Jiang et al., 2019 [26]	China	Cross-sectional	1149	10–12 y	49.0%	*FTO* rs9939609: TT, AT/AA	EBF in the first 4 mo (yes vs. no)	Sex, age, birth weight, method of birth delivery, weekday TV viewing, appetite, sleep, Tanner stage, parental BMI and education level	Triceps skinfold and subscapular skinfold thicknesses, BMI, and body fat percentage

Abbreviations: ALSPAC, Avon Longitudinal Study of Parents and Children; BF, breastfeeding; BMI, body mass index; EBF, exclusive breastfeeding; *FTO*, fat mass and obesity associated; GENDAI, Gene-Diet Attica Investigation; GENESIS, Growth, Exercise and Nutrition Epidemiological Study in Preschoolers; GRS, obesity-specific genetic risk score; *PPARG2*, peroxisome proliferator-activated receptor γ2; Ref., reference; SNP, single nucleotide polymorphism; WHR, waist-hip ratio.

Regarding the exposures of breastfeeding, 5 studies investigated the duration of any breastfeeding [[19], [20], [21], [22], [23]], and 4 studies investigated the duration of exclusive breastfeeding [[23], [24], [25], [26]], including 1 study that investigated the duration of both total breastfeeding and exclusive breastfeeding [23]. Seven studies investigated single genes [[19], [20], [21], [22],[24], [25], [26]], and 1 study investigated multiple genes [23]. Of the studies that investigated single genes, 5 investigated *FTO* [19,20,[24], [25], [26]], and 2 investigated peroxisome proliferator-activated receptor γ2 (*PPARG2* or *PPARγ2*) [21,22]. Seven studies reported BMI as the study outcome [[19], [20], [21],[23], [24], [25], [26]], 3 studies reported the outcome as skinfold thicknesses (comprising triceps skinfold and subscapular skinfold) [20,21,26]; 2 studies each reporting the outcome as age at adiposity peak and age at adiposity rebound [23,24], waist circumference [20,21], and BMI trajectory [23,25]; and only 1 study each reported the outcome as overweight/obesity [19], waist-hip ratio (WHR) [20], body fat percentage [26], and body weight growth rate [22]. Child age and sex were adjusted as confounders in half of studies [[19], [20], [21],^26^]. Other commonly adjusted confounders included maternal education, maternal BMI, smoking during pregnancy, household income, parity, and gestational age.

### Interaction association between genotypes and breastfeeding on weight status in children

**FIGURE 2:**
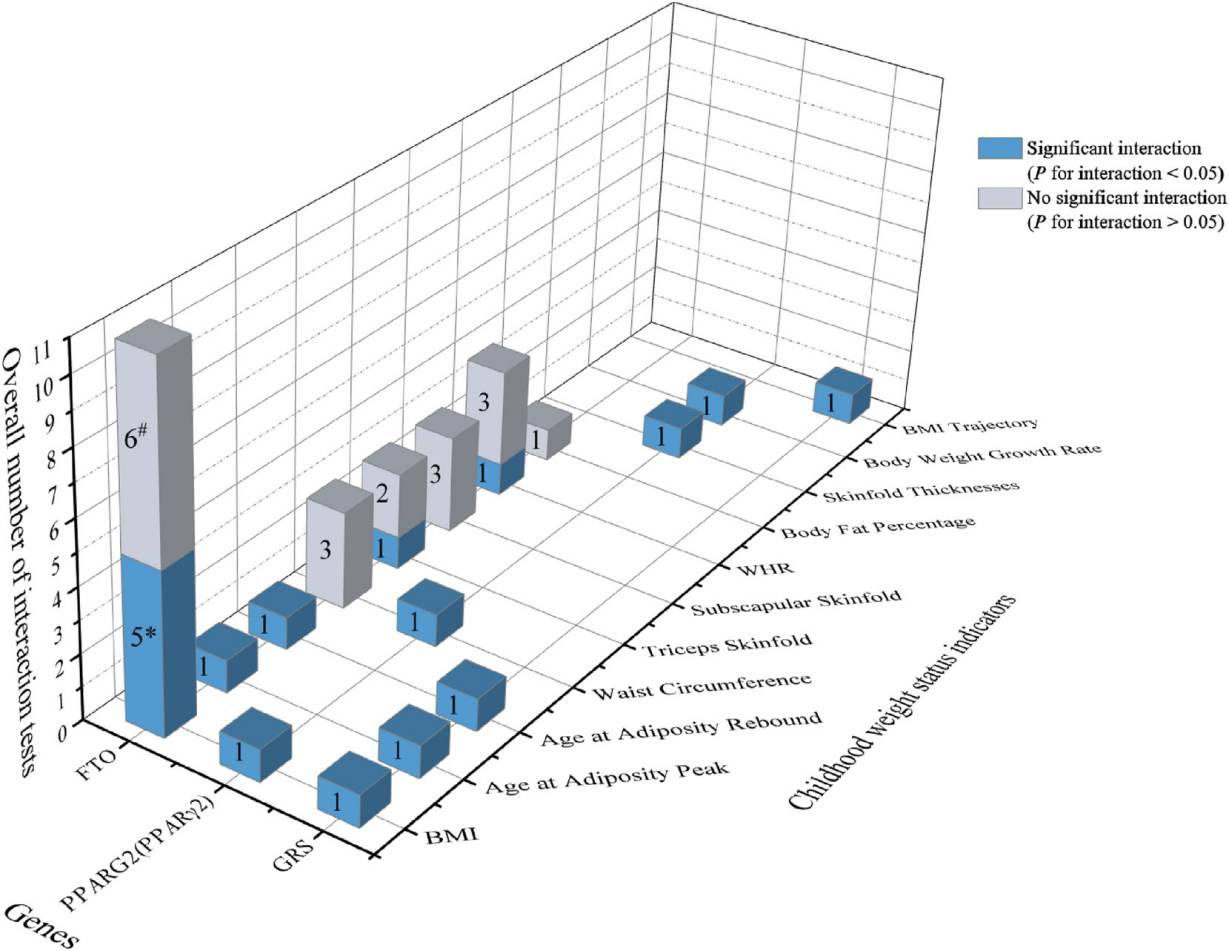
Significance of interaction terms between genotypes and breastfeeding/ exclusive breastfeeding on children’s weight-status indicators. Blue bars indicate significant interactions, for which effect the direction between breastfeeding and genes is antagonistic, and gray bars indicate no significant interactions. The number in each bar indicates the number of interaction tests. The study by Kanders et al. [19] analyzed 3 waves independently, and the study by Dedoussis et al. [20] analyzed 4 different cohorts independently, thus were separately counted. ∗One of the significant interaction tests by Abarin et al. [25] was only observed in girls. ^#^Indicates that one of nonsignificant interaction tests by Abarin et al. was only observed in boys. BMI, body mass index; *FTO*, fat mass and obesity associated; GRS, obesity-specific genetic risk score; *PPARG2*, peroxisome proliferator-activated receptor γ2; WHR, waist-hip ratio.

Thirty-five interaction tests between breastfeeding/exclusive breastfeeding and genotypes on children’s weight status indicators were examined among the 8 studies [[19], [20], [21], [22], [23], [24], [25], [26]], with 1 study analyzing 3 different waves of a cohort [19], 1 study analyzing 4 different cohorts independently [20], and another study analyzing girls and boys separately [25] and thus were all separately counted. Half the tests reported significant interactions between breastfeeding/exclusive breastfeeding and genotypes on children’s weight status indicators (*P* values for the interaction term < 0.05). The significance of interaction associations between breastfeeding and genotypes are shown on Figure 2. Longer breastfeeding durations were generally reported to attenuate the disadvantageous association between a risk allele of the genes and overdevelopment of children’s body weight, such as higher BMI [[19], [20], [21],[23], [24], [25]], WHR [20], and waist circumference [21] and earlier age at adiposity peak [23,24]. Nine of the 27 tests reported significant interactions of breastfeeding and *FTO* genotypes on children’s weight status indicators (*P* for interaction < 0.05) [19,20,24,25], including 1 significant interaction that was only observed among girls [25]. Four tests reported significant interactions of breastfeeding and *PPARG2* genotypes on children’s weight status indicators (*P* for interaction < 0.05) [21,22]. Another 4 tests showed significant interactions between breastfeeding and obesity-specific genetic risk score (GRS) (*P* for interaction < 0.05) [23].

### The combined association between genotypes and breastfeeding on child weight status

Among the 5 studies that examined the combined association between breastfeeding and genotypes on weight status in children [[19], [20], [21], [22], [23]], 2 investigated *FTO* [19,20], 2 investigated *PPARG2* [21,22], and 1 investigated a GRS consisting of 94 BMI-related single nucleotide polymorphism (SNPs) [23]. All 5 studies reported significant interactions between breastfeeding and genotypes (Table 2).

**TABLE 2:** The combined association between genotypes and breastfeeding on weight status in children.

Author, year, reference	Effect gene	Optimal duration of BF	Childhood weight status indicators	Combined association of genotypes and breastfeeding with weight status in children	Significant interaction effect	Nonsignificant interaction effect
Kanders et al., 2022 [19]	*FTO* rs9939609	>12 months BF vs. 0–6 months BF (Ref.)	Overweight/obesity; BMI	Wave 1 and wave 2: a longer duration of BF was negatively associated with children’s BMI among AA and TA carriers (adverse variant), although the association was positive among TT carriers Wave 1: each additional A allele (continuous variable) combined with BF for >12 mo, the odds of being overweight/obesity decreased 59% (OR: 0.41; 95% CI: 0.19, 0.88).	A longer BF duration attenuates the relationship between *FTO* rs9939609 adverse variant and BMI in wave 1 and wave 2 (interaction term *FTO* rs9939609 × BF in months: *P* for interaction < 0.05).	No statistically significant interaction effect between BF and *FTO* rs9939609 on BMI was found for wave 3 (Interaction term *FTO* rs9939609 × BF in months: *P* for interaction>0.05).
Dedoussis et al., 2011, [20]	GENDAI cohorts: *FTO* rs9939609 GENESIS cohorts: *FTO* rs17817449 ALSPAC cohorts: *FTO* rs9939609	Ever BF vs. no BF (Ref.)	BMI, waist circumference, WHR, triceps skinfold and subscapular skinfold	In non-BF children in the GENDAI cohort, carriers of the A allele had higher BMI, WHR, and waist circumference, whereas no differences in all obesity indices among the *FTO* genotype groups were observed in breastfed children.	Significant interactions were observed between BF and *FTO* genotypes on ≥1 obesity index in the 3 Greek cohorts (GENDAI: WHR; GENESIS cohorts in preschoolers aged 2–3 y: BMI; GENESIS cohorts aged 3–4 y: triceps skinfold). (*P* for interaction < 0.05).	No significant interaction was observed between BF and *FTO* genotypes in the following obesity indices in the British cohort (ALSPAC: BMI, WHR) and in the 3 Greek cohorts (GENDAI: BMI, waist circumference, triceps skinfold, subscapular skinfold; GENESIS cohorts in preschoolers aged 2–3 y: waist circumference, WHR, triceps skinfold, subscapular skinfold; GENESIS cohorts aged 3–4 y: BMI, waist circumference, WHR, subscapular skinfold) (*P* for interaction > 0.05).
Verier et al., 2010 [21]	*PPARG2*	BF vs. no BF (Ref.)	BMI, waist circumference, and skinfold thicknesses	Children who had not been BF: Ala12 allele (adverse variant) carriers had higher BMI (+1.88 kg/m^2^), higher waist circumference (3.8 cm), and higher skinfold thicknesses (16.3 mm) than Pro12Pro counterparts. Children who had been BF: no significant difference in adiposity measurements between Ala12 allele carriers and Pro12Pro counterparts.	BF appears to counter the deleterious effect of the *PPARG2* Pro12Ala polymorphism on anthropometric parameters including BMI, waist circumference and skinfold thicknesses in adolescents. (interaction term between *PPARG2* and BF, *P* for interaction < 0.05).	—
Mook-Kanamori et al., 2009 [22]	*PPARG2*	BF duration (mo): <2, 2–4, ≥4	Body weight growth rate	In infants who were BF for 4 mo, *PPARG2* Pro12Ala was not associated with growth rate. When BF duration was 2 mo or 2–4 mo, growth rate was higher in Ala12Ala than Pro12Pro carriers (differences 9.80 g/wk; 95% CI: 3.97, 15.63 and 6.32 g/wk; 95% CI: 1.04, 13.68, respectively).	Longer BF duration attenuates the association between *PPARγ2* adverse variant and body weight growth rate. A significant interaction was found between genotype and BF duration on children’s weight growth rate (*P* for interaction < 0.05).	—
Wu et al., 2020, [23]	A GRS of 94 SNPs, which represents the genetic susceptibility to overweight and obesity	Primary analysis: EBF duration (mo): 0 (Ref.), 3, 5 Secondary analysis: BF duration (mo): 0 (Ref.), 3, 5	BMI, BMI trajectory, age at AP, and age at AR	Primary analysis: EBF In the high genetic susceptible group (upper GRS quartile), EBF to 5 mo reduces BMI by 1.14 kg/m^2^ in 18-y-old boys, which compensates for a 3.9-decile GRS increase. In 18-y-old girls, EBF to 5 mo decreases BMI by 1.53 kg/m^2^ boys, which compensates for a 7.0-decile GRS increase. Effect of 5 mo EBF on timing of AP and AR by GRS levels: Age at AP: GRS 2.5 (delayed 0.17 y), GRS 5.0 (advanced 0.01 y), GRS 7.0 (delayed 0.25 y) in boys; GRS 2.5 (delayed 0.64 y), GRS 5.0 (delayed 0.12 y), GRS 7.0 (delayed 0.24 y) in girls. Age at AR: GRS 2.5 (delayed 0.64 y), GRS 5.0 (delayed 0.53 y), GRS 7.0 (delayed 0.44 y) in girls; GRS 2.5 (delayed 0.19 y), GRS 5.0 (delayed 0.18 y), GRS 7.0 (delayed 0.14 y) in boys. Secondary analysis: BF At 18 y, the reduction of BMI associated with 5 mo of BF varied in boys from 0.31–0.37 between the first and third GRS quartiles and from 0.34–0.54 in girls.	EBF attenuates the association between genetic susceptibility to BMI or timing of AP and AR. The study indicates a significant 3-way interaction between age, GRS, and EBF (or BF) in boys and girls (*P* for interaction < 0.05). Significant interactions were observed between BF and GRS on BMI trajectories (*P* for interaction < 0.05).	—
Wu et al., 2017 [24]	*FTO* rs9939609	5 mo EBF vs. no EBF (Ref.)	BMI, age at AP, and age at AR	BMI: EBF acts antagonistically to the *FTO* rs9939609 SNP A allele and compensates almost completely for the A allele effect among boys and girls. By age 15 y, the predicted reduction in BMI after 5 mo of EBF is 0.56 kg/m^2^ (95% CI: 0.11, 1.01) and 1.14 kg/m^2^ (95% CI: 0.67, 1.62) in boys and girls, respectively. Age at AP EBF effects (5 mo): TT: delayed 0.14 y in boys; delayed 0.22 y in girls. AT: delayed 0.19 y in boys; delayed 0.17 y in girls. AA: delayed 0.18 y in boys; delayed 0.23 y in girls. Age at AR EBF effects (5 mo): TT: delayed 0.10 y in boys; delayed 0.49 y in girls. AT: delayed 0.07 y in boys; delayed 0.52 y in girls. AA: delayed 0.08 y in boys; delayed 0.46 y in girls.	A longer duration of EBF (i.e., ≥5 mo) attenuates the increase in BMI and delays the timing of AP and AR among carriers of the risk allele of the *FTO* SNP rs9939609 (interaction term between *FTO* and EBF: *P* for interaction < 0.05).	—
Abarin et al., 2012 [25]	*FTO*	6 mo EBF vs. no EBF (Ref.)	BMI	In girls, EBF can reverse the increase in BMI because of *FTO* risk allele by age 14 y after 3 mo of EBF. In boys, EBF reduces BMI in carriers and noncarriers of the risk allele with an association found after 10 y of age. Six months of EBF restores boys’ BMI growth curves to the normal range.	In girls only, an interaction between EBF and the *FTO* genotypes was detected (*P* for interaction < 0.05), resulting in a substantial decrease in BMI of 0.119 kg/m^2^ for each month of EBF in the AT carriers and 0.180 kg/m^2^ in the AA carriers.	No interaction between EBF and *FTO* genotypes on BMI was found in boys (*P* for interaction > 0.05).
Jiang et al., 2019 [26]	*FTO rs9939609*	EBF in the first 4 mo (yes vs. no)	Triceps skinfold and subscapular skinfold thicknesses, BMI, body fat percentage	Carriers of *FTO* rs9939609 A allele had higher BMI and body fat percentage compared with non-A allele carriers, regardless of EBF in the first 4 mo.	—	No significant interactions were established between EBF in the first 4 mo and *FTO* rs9939609 on BMI or body fat percentage of children (*P* for interaction > 0.05).

Abbreviations: ALSPAC, Avon Longitudinal Study of Parents and Children; AP, adiposity peak; AR, adiposity rebound; BF, breastfeeding; BMI, body mass index; CI, confidence interval; EBF, exclusive breastfeeding; *FTO*, fat mass and obesity associated; GENDAI, Gene-Diet Attica Investigation; GENESIS, Growth, Exercise and Nutrition Epidemiological Study in Preschoolers; GRS, obesity-specific genetic risk score; *PPARG2*, peroxisome proliferator-activated receptor γ2; Ref., reference; SNP, single nucleotide polymorphism; WHR, waist-hip ratio.

In a Swedish cohort, Kanders et al. [19] reported breastfeeding as a moderator in the association between *FTO* rs9939609 genotypes and child BMI. In wave 1 (aged 14.4 y) and wave 2 (aged 17.3 y) assessments of the cohort, a longer duration of breastfeeding was negatively associated with children’s BMI among AA and TA carriers, although the association was positive among TT carriers (*P* for interaction < 0.05); no such interaction was found in the wave 3 assessment [19]. In a joint analysis of 3 pediatric cohorts, Dedoussis et al. [20] reported significant interactions between *FTO* genotypes and breastfeeding in 2 Greek cohorts (rs9939609 and rs17817449, respectively), but not in a British cohort (rs9939609) [20]. In one of the Greek cohorts, carriers of the A allele of (adverse variant) *FTO* rs9939609 in non-breastfed children had higher BMI, WHR, and waist circumference, whereas no differences in all obesity indices among the *FTO* genotype groups were observed in breastfed children [20]. *FTO* gene variants rs9939609 and rs17817449 have been reported to be in complete linkage disequilibrium (*r*^2^ = 1) in populations of European ancestry, indicating that rs17817449 is a perfect substitute for rs9939609; thus, these 2 SNPs are equivalent in terms of genetic effects [20].

In a multicenter cross-sectional study in Europe [21], among children who had not been breastfed, Ala12 allele (adverse variant) carriers of *PPARG2* had higher BMI (+1.88 kg/m^2^), higher waist circumference (3.8 cm), and higher skinfold thicknesses (16.3 mm) than their Pro12Pro counterparts, at mean age of 14.7 y. However, among children who had ever been breastfed, there was no significant difference in adiposity measurements between Ala12 allele carriers and their Pro12Pro counterparts, regardless of the breastfeeding duration. In a Dutch cohort [22], *PPARG2* Pro12Ala was not associated with body weight growth rate among infants who were breastfed for 4 mo; when breastfeeding duration was <4 mo, growth rate was higher in Ala12Ala than in Pro12Pro carriers.

In a UK cohort, Wu et al. [23] calculated the GRS of 94 SNPs, which represents the genetic susceptibility to overweight and obesity. At 18 y, the reduction of BMI associated with 5 mo of breastfeeding varied in boys from 0.31 kg/m^2^ to 0.37 kg/m^2^ between the first and third GRS quartiles and from 0.34 kg/m^2^ to 0.54 kg/m^2^ in girls.

### The combined association between genotypes and exclusive breastfeeding on child weight status

Three studies reported combined associations between a single gene (*FTO*) and exclusive breastfeeding on child weight status [[24], [25], [26]]. Furthermore, one study reported the combined association between multiple genes and exclusive breastfeeding on weight status in children [23]. Three of the 4 studies reported significant interactions between exclusive breastfeeding and genotypes, which indicated exclusive breastfeeding somewhat antagonizes obesity genes and lowers body weight indicators in children [[23], [24], [25]] (Table 2).

In a UK cohort, a longer duration of exclusive breastfeeding (i.e., ≥5 mo) attenuated the increase in BMI and delayed the timing of the adiposity peak and adiposity rebound among carriers of the risk allele of *FTO* rs9939609 (*P* for interaction < 0.05) [24]; exclusive breastfeeding acts antagonistically to the *FTO* rs9939609 A allele and compensates almost completely the A allele effect in boys and girls. In an Australian cohort, a significant interaction between exclusive breastfeeding and the *FTO* rs9939609 genotype was detected only in girls (*P* for interaction < 0.05), resulting in a substantial decrease in BMI of 0.119 kg/m^2^ for each month of breastfeeding in the AT carriers and 0.180 kg/m^2^ in the AA carriers [25]. In a multiple-gene study, in the high genetic susceptible group (upper GRS quartile), exclusive breastfeeding to 5 mo reduced BMI by 1.14 kg/m^2^ in 18-y-old boys, which compensated for a 3.9-decile GRS increase, and in girls, decreased BMI by 1.53 kg/m^2^, which compensated for a 7.0-decile GRS increase [23]. However, one study showed no significant interaction between exclusive breastfeeding in the first 4 mo and the *FTO* rs9939609 on BMI or body fat percentage in Chinese children (*P* for interaction > 0.05) [26]. In the study, carriers of *FTO* rs9939609 A allele had higher BMI and body fat percentage compared with non-A allele carriers, regardless of whether they had been exclusively breastfed or not [26].

### Dose-response relationship of exclusive breastfeeding duration with children’s weight status and comparison of the effect of exclusive breastfeeding compared with nonexclusive breastfeeding

One study examined the dose-response relationship of exclusive breastfeeding duration with children’s weight status at 7, 10, 15, and 18 y of age for different GRSs and reported a longer time of exclusive breastfeeding was associated with larger decreases in BMI, regardless of the GRS [23]. The study also compared 5 mo of exclusive breastfeeding with nonexclusive breastfeeding and their association with BMI measurements. Generally, exclusive breastfeeding was associated with larger decrease in BMI growth trajectories compared to exclusive breastfeeding, regardless of the GRS level.

### Risk of bias

The 8 studies included in this systematic review were evaluated for the risk of bias and overall quality using the NOS and AHRQ methodological checklists. They were subsequently rated as high-quality. In the NOS methodological checklist, 3 studies scored 9 [[23], [24], [25]], 2 scored 8 [19,20], and 1 scored 6 [22]. In the AHRQ methodological checklist, one study scored 10 [21] and another scored 9 [26]. The specific risks of bias are reported in Supplementary Tables 5 and 6.

## Discussion

To our knowledge, this is the first systematic review to assess the interaction association between genetic status and breastfeeding on child weight status. *FTO* and *PPARG2* were the most examined genes. Only a few studies showed a significant interaction between breastfeeding and genotypes (*PPARG2* and GRS) on child weight status, although the evidence for an interaction between breastfeeding and *FTO* genotypes on child weight status was inconclusive and depended on different indicators of child weight status. Among studies reporting significant interactions, breastfeeding or exclusive breastfeeding was generally reported to attenuate the disadvantageous association between carrying a risk allele of the genes and overdevelopment of children’s body weight.

Previous systematic reviews have demonstrated the role of breastfeeding and the specific nutrients in human milk on child body weight and growth [[27], [28], [29]]. Although mixed findings were reported, most studies have demonstrated significant associations between longer duration of breastfeeding and lower BMI among children. Hormones in human milk, such as leptin and adiponectin, were reported to have inverse associations with child overgrowth of body weight and may explain the protective association of breastfeeding. However, the previous review studies failed to evaluate the association between breastfeeding and child weight status among children with different genetic predispositions, which may explain the inconsistent findings and help identify the target population for breastfeeding intervention [[27], [28], [29]].

Single-gene obesity studies have laid the foundation for childhood obesity research, and >100 loci have been confirmed to be highly associated with the development of obesity, including the *FTO* and *PPARG2* genes [[30], [31], [32]]. Of these, the *FTO* gene has the greatest impact on obesity [33]. Although the significance of the interaction between the *FTO* gene and breastfeeding is not consistent among different studies, depending on childhood weight status indicators, some studies suggest that breastfeeding has a modifying association with *FTO*, leading to childhood obesity [19,20,24,25]. The specific duration of breastfeeding that is most effective for the effect on the *FTO* gene has not been determined; however, studies have found that the longer the duration of breastfeeding (≥12 mo), the better the association of breastfeeding on the modification of the *FTO* gene, which causes obesity in children. Exclusive breastfeeding for ≥6 mo has also been recommended. The biological role of breastfeeding remains ambiguous; evidence suggests an association between breast milk signaling and *FTO*-activated transcription in breast milk recipients [34]. Liu et al. [35] reported that the hypomethylation of specific cytosine phosphate guanine sites in *FTO* enhances *FTO* expression. Breast milk may also alter DNA methylation through the transfer of milk-derived exosomes, which function similarly to epigenetic transfection systems [36]. The specific mechanism may be that breast milk mediates miRNA-29b/miRNA-21/DNMT signaling, which fosters the demethylation of CpG sites within the *FTO* intron 1 [34]. This process upregulates *FTO* expression, which plays a vital role as an amplifier of the transcriptional machinery essential for postnatal growth. Another possible explanation is that *FTO* rs9939609 AA is phenotypically plastic [19] and manifests different phenotypes in disparate environmental contexts and that the expression of this genotype is affected by different environmental factors [37,38]. Breast milk is a dynamic substance comprising a multitude of nutrients, continuously evolving in response to the changing needs of infants at various stages of growth [39]. During feeding, the composition of breast milk undergoes a complex and dynamic transformation to meet the requirements of each infant [40]. Breastfeeding supports better energy balance during the critical period of infant development by providing the optimal mix of nutrients. This balanced diet helps regulate energy intake, preventing rapid weight gain and reducing the risk of obesity, which may be influenced by the effects of the *FTO* gene [25]. However, these mechanisms were only inferred, and further research is needed to clarify the effect of breastfeeding on the association between *FTO* and childhood obesity.

Phenotypes modulated by *PPARG2* polymorphisms can be influenced by gene–environment interactions in early life [21]. How breastfeeding may counteract the deleterious effect of the Ala12 allele in adolescents may be explained by a number of mechanisms. There has been evidence that the association between dietary fat and BMI may be influenced by *PPARG2* genotypes. Memisoglu et al. [41] found that a diet high in monounsaturated fatty acids was inversely associated with BMI in carriers of the Ala12 allele but did not find an association in Pro12Pro women. Similarly, Luan et al. [42] reported that when a diet with a low ratio of polyunsaturated to saturated fatty acids was consumed, Ala12 allele carriers had a higher BMI than Pro12Pro carriers. Breast milk represents a diet with a specific fat intake with a higher proportion of polyunsaturated fatty acids than formula milk [43]. Another possible hypothesis is that breast milk or breastfeeding provides factors such as prostaglandin J2 [44], a natural *PPARG2* ligand. Breast milk may therefore compensate for the decrease in *PPARG2* transcriptional activity observed in Ala12 allele carriers. A number of adipokines are also present in the latter. It is known that *PPARG2* agonists (e.g., thiazolidinediones) can downregulate leptin expression [45]; however, the presence of this compound in breast and/or formula milk remains to be determined and would need to be studied in more detail.

Due to the limited impact of single gene mutations on the pathogenesis of obesity, the single obesity-causing genes identified so far explain <5% of severe obesity [46]. Genetic studies on obesity and methods of identifying susceptibility genes are continuing to evolve and progress, and several studies have found that obesity is inherited through multiple genes [32]; therefore, it is essential to conduct polygenic studies. There is also a need to investigate the role of breastfeeding in the modification of the polygenic association with childhood obesity.

From the perspective of public health, the prevalence of exclusive breastfeeding can be critical in charting a trajectory toward a healthy life course, as breast milk is the first source of nourishment for all infants at birth and is now widely acknowledged to be necessary for the optimal growth and development of infants [34]. Wu et al. [23] reported that 5 mo of exclusive breastfeeding had more impact on the association between genes and BMI than exclusive breastfeeding for 3 mo, which demonstrates a strong dose-response to continued exclusive breastfeeding. These findings support the recommendation of the WHO on the significance of exclusive breastfeeding for 6 mo. Regarding the role of the optimal duration of exclusive breastfeeding in the modification of the *FTO* gene associated with childhood obesity, Wu et al. [24] and Abarin et al. [25] reported different results. Wu et al. reported an optimal duration of exclusive breastfeeding of ≥5 mo, whereas Abarin et al. indicated that in boys, exclusive breastfeeding for ≥6 mo and in girls, no <3 mo of exclusive breastfeeding would be sufficient. There are few existing studies examining threshold values of both duration and dose of breastfeeding and their role in preventing childhood obesity among different gene variant carriers. Further research is needed to understand the biological mechanisms of the interaction association between breastfeeding and genes with childhood obesity and to compare the differences in modification association between different doses of breastfeeding. In addition, 7 of the 8 studies were in Western populations and reported significant interactions between breastfeeding and genetics, although the only study conducted in China failed to observe a significant interaction. Because there are some genetic differences between the races [47], subsequent studies of non-European populations should be conducted to further examine the race/ethnicity difference in the association. One study reported that significant interactions between exclusive breastfeeding and *FTO* genotype on children’s weight were only detected among girls, which indicated there may be a sex difference in the interaction association, although more evidence is still needed [25].

### Strengths and limitations

This systematic review has several strengths. A professional search strategy was developed and searched following PRISMA [13]. To increase the rigor of the reviews and reduce subjectivity, full-text reviews were conducted by 3 reviewers. Prospective studies are less prone to certain types of bias and are more likely to demonstrate that exposure precedes disease, thus making a stronger case of causation [48]. Therefore, another strength is the inclusion of mainly prospective cohort studies (6 of 8) rather than retrospective studies. Individual studies were considered high-quality because they used validated measures, had appropriate study designs, and had long follow-up periods.

This systematic review has some limitations. Primarily, the number of studies on the interaction association between genotypes and breastfeeding in childhood obesity is limited; thus, this systematic review cannot be aggregated and analyzed on a large scale. Second, the currently available studies were based on observational studies and lacked randomized controlled trials. Third, interpreting the evidence is challenging because of the different outcomes and effect measures used in the 8 studies. Furthermore, the studies included in this systematic review differed in their categorization of breastfeeding duration. Regarding children, variations in the results of the different body composition measures in the selected studies are another limitation, as it limits the comparability between studies. Finally, there was heterogeneity in the study variables, such as breastfeeding classification and gene selection, making it difficult to perform a meta-analysis of the data.

### Implications and recommendations

Early studies mainly explored obesity-causing genes from a single-gene perspective, and >20 causative genes have been confirmed to be highly associated with the development of obesity, including *FTO*, *PPARG2*, *LEP*, *MC4R,* and *POMC* [30]. In this systematic review, the types of genes studied in single-gene studies were few and only included *FTO* and *PPARG2*, which suggests a gap in the literature. Future studies should broaden their scope to include a wider array of genes, such as *MC4R*, *LEP*, and *POMC*, to provide a more comprehensive understanding of the combined association between these genes and breastfeeding and their association with obesity. Moreover, the review highlights the need for increased attention to polygenic risk factors, given that obesity is a polygenic disease. Future research should delve into the effects of breastfeeding on overall DNA methylation and polygenic risks, which could offer new insights into the complex interplay between genetics and breastfeeding. To date, randomized controlled trials to demonstrate causal relationships are lacking in this research field, and further studies are needed. In addition, as an alternative method to prove causality, Mendelian randomization (MR) uses genetic variants as instrumental variables to infer causality and can effectively address confounders and reverse causality in observational studies [49]. Using MR, analysis of the relationship between breastfeeding-related genetic variation and obesity outcomes may provide a practical alternative to randomized controlled trials and evidence for a causal relationship between breastfeeding and obesity.

## Conclusions

In conclusion, although the results of interactions between breastfeeding and genotypes on children’s weight status are inconsistent, some of studies have reported that breastfeeding or exclusive breastfeeding may attenuate the disadvantageous association between genetic risk alleles of the genes and overdevelopment of children’s body weight. These findings support the WHO recommendations for prolonged breastfeeding, and further suggested breastfeeding interventions to prevent childhood obesity may be more effective in populations with a disadvantageous genetic predisposition.

## Author contributions

The authors’ responsibilities were as follows – ZY, JJH: designed the research; ZY, LZ, XW: conducted the literature search; ZY, NL, HC, JJH: performed study screening, data extraction, and study quality assessment; ZY: wrote the manuscript; NL, HC, BRL, JJH: contributed to writing the discussion; XCW, LZ, XYY, SQW: contributed to interpretation of the study findings; ZY, NL, HC, BRL, XCW, LZ, XYY, SQW, JJH, DLW: provided critical review and contributed to the manuscript; and all authors: read and approved the final manuscript.

## Conflicts of interest

The authors report no conflicts of interest.

## Funding

This review was supported by the following grants: the National Natural Science Foundation of China (82103860); the 111 project (grant number D21008); National Key Research and Development Program of China (No. 2021YFC2701500) and the Education Department of Liaoning Province (JYTQN2023035).

## Data availability

The data used in this review will be made available from the corresponding author upon written request.
