# Supplementary material for: Influence of the Interaction between Genetic Factors and Breastfeeding on Children’s Weight Status: A Systematic Review
Source: Adv Nutr. 2024 Oct 9;15(11):100312. doi: 10.1016/j.advnut.2024.100312 (PMC11566687; doi:10.1016/j.advnut.2024.100312)
Supplement: Multimedia component 1 [file mmc1.docx]

# Supplementary materials

# Supplementary Table 1. Search strategy in the PubMed database, from inception to August 12, 2024

| **No.** | **Num** | **Searches** |
| --- | --- | --- |
| 1 | 108,741 | "breast feeding"[MeSH] OR "Milk, Human"[Mesh] OR "Colostrum"[Mesh] OR "Lactation"[Mesh] |
| 2 | 287,299 | breastfe*[Title/Abstract] OR "breast fed"[Title/Abstract] OR "breast feed"[Title/Abstract] OR "breast milk"[Title/Abstract] OR "Human Milk"[Title/Abstract] OR lactat*[Title/Abstract] OR "infant feeding"[Title/Abstract] OR breastfed[Title/Abstract] OR breastmilk[Title/Abstract] OR Colostrum[Title/Abstract] OR foremilk[Title/Abstract] OR hindmilk[Title/Abstract] OR ((human[Title/Abstract] OR breast*[Title/Abstract] OR mother*[Title/Abstract] OR MOM[Title/Abstract] OR expressed[Title/Abstract] OR maternal[Title/Abstract] OR donor*[Title/Abstract]) AND (milk*[Title/Abstract] OR breastmilk*[Title/Abstract])) |
| 3 | 293,092 | "Adiposity"[Mesh] OR "Overweight"[Mesh] OR "Obesity"[Mesh] |
| 4 | 301,831 | "Body Fat Distribution"[Majr] OR "Body Composition"[Majr] OR "Body Mass Index"[Majr] OR "Body Weight"[Majr] OR "Waist Circumference"[Majr] OR "Waist-Height Ratio"[Majr] OR "Skinfold Thickness"[Majr] OR "Waist-Hip Ratio"[Majr] OR "Body Weight Changes"[Majr] OR "Abdominal Fat"[Majr] OR "Weight Reduction Programs"[Majr] OR "Overnutrition"[Majr] OR "Body Size"[Majr:NoExp] OR "Body Weights and Measures"[Majr:NoExp] |
| 5 | 1,299,085 | obes*[Title/Abstract] OR adipos*[Title/Abstract] OR "over weight"[Title/Abstract] OR overweight[Title/Abstract] OR overeat*[Title/Abstract] OR "over eat*"[Title/Abstract] OR overfeed*[Title/Abstract] OR "over feed*"[Title/Abstract] OR overnourish*[Title/Abstract] OR "over‐nourish*"[Title/Abstract] OR "overnutrit*"[Title/Abstract] OR "over‐nutrit*"[Title/Abstract] OR "overload syndrome*"[Title/Abstract] OR fat[Title/Abstract] OR "body mass index"[Title/Abstract] OR body-mass*[Title/Abstract] OR "body mass"[Title/Abstract] OR BMI[Title/Abstract] OR "weight status"[Title/Abstract] OR bodyweight[Title/Abstract] OR "body weight"[Title/Abstract] OR "body size"[Title/Abstract] OR "body composition"[Title/Abstract] OR "weight gain"[Title/Abstract] OR "weight cycling"[Title/Abstract] OR "weight change*"[Title/Abstract] OR "weight reduc*"[Title/Abstract] OR "weight los*"[Title/Abstract] OR "weight maint*"[Title/Abstract] OR "weight decreas*"[Title/Abstract] OR "weight watch*"[Title/Abstract] OR "weight control*"[Title/Abstract] OR "weight modif*"[Title/Abstract] OR "skinfold thickness"[Title/Abstract] OR "abdominal circumference"[Title/Abstract] OR "waist circumference"[Title/Abstract] OR "waist hip ratio"[Title/Abstract] OR "waist-hip ratio"[Title/Abstract] OR WHR[Title/Abstract] OR "waist height ratio"[Title/Abstract] OR "waist-height ratio"[Title/Abstract] OR WHtR[Title/Abstract] |
| 6 | 1,133,066 | "epigenome"[MeSH] OR "genes"[MeSH] OR "Genetics"[Mesh] |
| 7 | 6,738,746 | epigenom*[Title/Abstract] OR epigenetic*[Title/Abstract] OR genetic[Title/Abstract] OR gene*[Title/Abstract] OR Polygenic*[Title/Abstract] OR familial[Title/Abstract] or inherit*[Title/Abstract] or heredit*[Title/Abstract] |
| 8 | 4,076,049 | "Child"[Mesh] OR "Adolescent"[MeSH] OR "infant"[MeSH] |
| 9 | 2,825,581 | adolescence[Title/Abstract] OR teen*[Title/Abstract] OR child*[Title/Abstract] OR adolescen*[Title/Abstract] OR offspring[Title/Abstract] OR infan*[Title/Abstract] OR newborn[Title/Abstract] OR new-born[Title/Abstract] OR neonat*[Title/Abstract] OR "new born"[Title/Abstract] OR "new borns"[Title/Abstract] OR "newly born"[Title/Abstract] OR baby*[Title/Abstract] OR babies[Title/Abstract] OR premature[Title/Abstract] OR premie[Title/Abstract] OR premies[Title/Abstract] OR prematurity[Title/Abstract] OR preterm[Title/Abstract] OR "pre term"[Title/Abstract] OR toddler*[Title/Abstract] |
| 10 | 3,387 | (#1 OR #2) AND (#3 OR #4 OR #5) AND (#6 OR #7) AND (#8 OR #9) |

# Supplementary Table 2. Search strategy in the Web of Science database, from inception to August 12, 2024

| **No.** | **Num** | **Searches** |
| --- | --- | --- |
| 1 | 190,301 | TS= ("breast feeding" OR "Milk, Human" OR Colostrum OR Lactation) |
| 2 | 372,984 | TI= (breastfe* OR "breast fed" OR "breast feed" OR "breast milk" OR "Human Milk" OR lactat* OR "infant feeding" OR breastfed OR breastmilk OR Colostrum OR foremilk OR hindmilk) OR AB= (breastfe* OR "breast fed" OR "breast feed" OR "breast milk" OR "Human Milk" OR lactat* OR "infant feeding" OR breastfed OR breastmilk OR Colostrum OR foremilk OR hindmilk) |
| 3 | 87,862 | (TI= (human OR breast* OR mother* OR MOM OR expressed OR maternal OR donor*) OR AB= (human OR breast* OR mother* OR MOM OR expressed OR maternal OR donor*)) AND (TI= (milk* OR breastmilk*) OR AB= (milk* OR breastmilk*)) |
| 4 | 854,625 | TS= (Adiposity OR Overweight OR Obesity) |
| 5 | 1,236,445 | TS= ("Body Fat Distribution" OR "Body Composition" OR "Body Mass Index" OR "Body Weight" OR "Waist Circumference" OR "Waist-Height Ratio" OR "Skinfold Thickness" OR "Waist-Hip Ratio" OR "Body Weight Changes" OR "Abdominal Fat" OR "Weight Reduction Programs" OR "Overnutrition" OR "Body Size" OR "Body Weights and Measures") |
| 6 | 596,010 | TI= (obes* OR adipos* OR "over weight" OR overweight OR overeat* OR "over eat*" OR overfeed* OR "over feed*" OR overnourish* OR "over-nourish*" OR "overnutrit*" OR "over-nutrit*" OR "overload syndrome*" OR fat OR "body mass index" OR body-mass* OR "body mass" OR BMI OR "weight status" OR bodyweight OR "body weight" OR "body size" OR "body composition" OR "weight gain" OR "weight cycling" OR "weight change*" OR "weight reduc*" OR "weight los*" OR "weight maint*" OR "weight decreas*" OR "weight watch*" OR "weight control*" OR "weight modif*" OR "skinfold thickness" OR "abdominal circumference" OR "waist circumference" OR "waist hip ratio" OR "waist-hip ratio" OR WHR OR "waist height ratio" OR "waist-height ratio" OR WHtR) |
| 7 | 1,804,249 | AB= (obes* OR adipos* OR "over weight" OR overweight OR overeat* OR "over eat*" OR overfeed* OR "over feed*" OR overnourish* OR "over‐nourish*" OR "overnutrit*" OR "over‐nutrit*" OR "overload syndrome*" OR fat OR "body mass index" OR body-mass* OR "body mass" OR BMI OR "weight status" OR bodyweight OR "body weight" OR "body size" OR "body composition" OR "weight gain" OR "weight cycling" OR "weight change*" OR "weight reduc*" OR "weight los*" OR "weight maint*" OR "weight decreas*" OR "weight watch*" OR "weight control*" OR "weight modif*" OR "skinfold thickness" OR "abdominal circumference" OR "waist circumference" OR "waist hip ratio" OR "waist-hip ratio" OR WHR OR "waist height ratio" OR "waist-height ratio" OR WHtR) |
| 8 | 8,778,811 | TS= (epigenome OR genes OR Genetics) |
| 9 | 13,904,425 | TI= (epigenom* OR epigenetic* OR genetic OR gene* OR Polygenic* OR familial or inherit* or heredit*) OR AB= (epigenom* OR epigenetic* OR genetic OR gene* OR Polygenic* OR familial or inherit* or heredit*) |
| 10 | 6,234,854 | TS= (child OR adolescent OR infant) |
| 11 | 4,208,905 | TI= (adolescence OR teen* OR child* OR adolescen* OR offspring OR infan* OR newborn OR new-born OR neonat* OR "new born" OR "new borns" OR "newly born" OR baby* OR babies OR premature OR premie OR premies OR prematurity OR preterm OR "pre term" OR toddler*) OR AB= (adolescence OR teen* OR child* OR adolescen* OR offspring OR infan* OR newborn OR new-born OR neonat* OR "new born" OR "new borns" OR "newly born" OR baby* OR babies OR premature OR premie OR premies OR prematurity OR preterm OR "pre term" OR toddler*) |
| 12 | 7,444 | (#1 OR (#2 OR#3)) AND (#4 OR #5 OR (#6 OR #7)) AND (#8 OR #9) AND (#10 OR #11) |

# Supplementary Table 3. Search strategy in the Embase database, from inception to August 12, 2024

| **No.** | **Num** | **Searches** |
| --- | --- | --- |
| 1 | 160,746 | 'breast feeding'/exp OR 'breast milk'/exp OR 'colostrum'/exp OR 'Lactation'/exp |
| 2 | 350,127 | breastfe*:ab,ti OR 'breast fed':ab,ti OR 'breast feed':ab,ti OR 'breast milk':ab,ti OR 'human milk':ab,ti OR lactat*:ab,ti OR 'infant feeding':ab,ti OR breastfed:ab,ti OR breastmilk:ab,ti OR colostrum:ab,ti OR foremilk:ab,ti OR hindmilk:ab,ti OR ((human:ab,ti OR breast*:ab,ti OR mother*:ab,ti OR mom:ab,ti OR expressed:ab,ti OR maternal:ab,ti OR donor*:ab,ti) AND (milk*:ab,ti OR breastmilk*:ab,ti)) |
| 3 | 2,089,306 | 'adiposity'/exp OR 'overweight'/exp OR 'obesity'/exp OR 'body fat distribution'/exp OR 'body composition'/exp OR 'body mass'/exp OR 'body weight'/exp OR 'waist circumference'/exp OR 'waist to height ratio'/exp OR 'skinfold thickness'/exp OR 'waist hip ratio'/exp OR 'body weight change'/exp OR 'abdominal fat'/exp OR 'weight loss program'/exp OR 'overnutrition'/exp OR 'body size'/exp OR 'morphometry'/exp |
| 4 | 1,890,577 | obes*:ab,ti OR adipos*:ab,ti OR 'over weight':ab,ti OR overweight:ab,ti OR overeat*:ab,ti OR 'over eat*':ab,ti OR overfeed*:ab,ti OR 'over feed*':ab,ti OR overnourish*:ab,ti OR 'over-nourish*':ab,ti OR 'overnutrit*':ab,ti OR 'over-nutrit*':ab,ti OR 'overload syndrome*':ab,ti OR fat:ab,ti OR 'body mass index':ab,ti OR 'body mass*':ab,ti OR 'body mass':ab,ti OR bmi:ab,ti OR 'weight status':ab,ti OR bodyweight:ab,ti OR 'body weight':ab,ti OR 'body size':ab,ti OR 'body composition':ab,ti OR 'weight gain':ab,ti OR 'weight cycling':ab,ti OR 'weight change*':ab,ti OR 'weight reduc*':ab,ti OR 'weight los*':ab,ti OR 'weight maint*':ab,ti OR 'weight decreas*':ab,ti OR 'weight watch*':ab,ti OR 'weight control*':ab,ti OR 'weight modif*':ab,ti OR 'skinfold thickness':ab,ti OR 'abdominal circumference':ab,ti OR 'waist circumference':ab,ti OR 'waist hip ratio':ab,ti OR 'waist-hip ratio':ab,ti OR whr:ab,ti OR 'waist height ratio':ab,ti OR 'waist-height ratio':ab,ti OR whtr:ab,ti |
| 5 | 2,542,845 | 'epigenome'/exp OR 'genes'/exp OR 'genetics'/exp |
| 6 | 8,327,421 | epigenom*:ab,ti OR epigenetic*:ab,ti OR genetic:ab,ti OR gene*:ab,ti OR polygenic*:ab,ti OR familial:ab,ti OR inherit*:ab,ti OR heredit*:ab,ti |
| 7 | 4,485,095 | 'child'/exp OR 'adolescent'/exp OR 'infant'/exp |
| 8 | 3,613,317 | adolescence:ab,ti OR teen*:ab,ti OR child*:ab,ti OR adolescen*:ab,ti OR offspring:ab,ti OR infan*:ab,ti OR newborn:ab,ti OR neonat*:ab,ti OR 'new born':ab,ti OR 'new borns':ab,ti OR 'newly born':ab,ti OR baby*:ab,ti OR babies:ab,ti OR premature:ab,ti OR premie:ab,ti OR premies:ab,ti OR prematurity:ab,ti OR preterm:ab,ti OR 'pre term':ab,ti OR toddler*:ab,ti |
| 9 | 7,242 | (#1 OR #2) AND (#3 OR #4) AND (#5 OR #6) AND (#7 OR #8) |

# Supplementary Table 4. Search strategy in the Cochrane database, from inception to August 12, 2024

| **No.** | **Num** | **Searches** |
| --- | --- | --- |
| 1 | 2,935 | MeSH descriptor: [Breast Feeding] explode all trees |
| 2 | 1,526 | MeSH descriptor: [Milk, Human] explode all trees |
| 3 | 2,16 | MeSH descriptor: [Colostrum] explode all trees |
| 4 | 4,067 | #1 OR #2 OR #3 |
| 5 | 31,784 | (breastfe* OR "breast fed" OR "breast feed" OR "breast milk" OR "Human Milk" OR lactat* OR "infant feeding" OR breastfed OR breastmilk OR Colostrum OR foremilk OR hindmilk):ti,ab,kw |
| 6 | 9,582 | (human OR breast* OR mother* OR MOM OR expressed OR maternal OR donor*):ti,ab,kw AND (milk* OR breastmilk*):ti,ab,kw |
| 7 | 1,129 | MeSH descript: [Adiposity] explode all trees |
| 8 | 25,261 | MeSH descript: [Overweight] explode all trees |
| 9 | 21,563 | MeSH descript: [Obesity] explode all trees |
| 10 | 1,361 | MeSH descript: [Body Fat Distribution] explode all trees |
| 11 | 7,207 | MeSH descript: [Body Composition] explode all trees |
| 12 | 14,128 | MeSH descript: [Body Mass Index] explode all trees |
| 13 | 40,767 | MeSH descript: [Body Weight] explode all trees |
| 14 | 1,480 | MeSH descript: [Waist Circumference] explode all trees |
| 15 | 22 | MeSH descript: [Waist-Height Ratio] explode all trees |
| 16 | 389 | MeSH descript: [Skinfold Thickness] explode all trees |
| 17 | 350 | MeSH descript: [Waist-Hip Ratio] explode all trees |
| 18 | 12,538 | MeSH descript: [Body Weight Changes] explode all trees |
| 19 | 724 | MeSH descript: [Abdominal Fat] explode all trees |
| 20 | 1,149 | MeSH descript: [Weight Reduction Programs] explode all trees |
| 21 | 25,278 | MeSH descript: [Overnutrition] explode all trees |
| 22 | 251 | MeSH descript: [Body Size] this term only |
| 23 | 316 | MeSH descript: [Body Weights and Measures] this term only |
| 24 | 50,830 | #7 OR #8 OR #9 OR #10 OR #11 OR #12 OR #13 OR #13 OR #15 OR #16 OR #17 OR #18 OR #19 OR #20 OR #21 OR #22 OR #23 |
| 25 | 211,113 | (obes* OR adipos* OR "over weight" OR overweight OR overeat* OR (over NEXT eat*) OR overfeed* OR (over NEXT feed*) OR overnourish* OR (over NEXT nourish*) OR overnutrit* OR (over NEXT nutrit*) OR (overload NEXT syndrome*) OR fat OR "body mass index" OR (body NEXT mass*) OR "body mass" OR BMI OR "weight status" OR bodyweight OR "body weight" OR "body size" OR "body composition" OR "weight gain" OR "weight cycling" OR (weight NEXT change*) OR (weight NEXT reduc*) OR (weight NEXT los*) OR (weight NEXT maint*) OR (weight NEXT decreas*) OR (weight NEXT watch*) OR (weight NEXT control*) OR (weight NEXT modif*) OR "skinfold thickness" OR "abdominal circumference" OR "waist circumference" OR "waist hip ratio" OR "waist-hip ratio" OR WHR OR "waist height ratio" OR "waist-height ratio" OR WHtR):ti,ab,kw |
| 26 | 2,522 | MeSH descriptor: [Genes] explode all trees |
| 27 | 281 | MeSH descriptor: [Epigenesis, Genetic] explode all trees |
| 28 | 28 | MeSH descriptor: [Epigenomics] explode all trees |
| 29 | 2,531 | #26 OR #27 #28 |
| 30 | 312,898 | (epigenom* OR epigenetic* OR genetic OR gene* OR Polygenic* OR familial or inherit* or heredit*):ti,ab,kw |
| 31 | 82,499 | MeSH descriptor: [Child] explode all trees |
| 32 | 137,774 | MeSH descriptor: [Adolescent] explode all trees |
| 33 | 46,409 | MeSH descriptor: [infant] explode all trees |
| 34 | 83,688 | #31 OR #32 #33 |
| 35 | 381,526 | (adolescence OR teen* OR child* OR adolescen* OR offspring OR infan* OR newborn OR new-born OR neonat* OR "new born" OR "new borns" OR "newly born" OR baby* OR babies OR premature OR premie OR premies OR prematurity OR preterm OR "pre term" OR toddler*):ti,ab,kw |
| 36 | 63 | (#4 OR (#5 OR #6)) AND (#24 OR #25) AND (#29 OR #30) AND (#34 OR #34) |

# Supplementary Table 5. Quality assessments of included cohort studies

| Items | | Kanders et al.  (2022) (19) | Dedoussis et al.  (2011) (20) | Mook-Kanamori et al.  (2009) (22) | Wu et al.  (2020) (23) | Wu et al.  (2017) (24) | Abarin et al.  (2012) (25) |
| --- | --- | --- | --- | --- | --- | --- | --- |
| Selection | Representativeness of the exposed cohort | ★ | ★ | ★ | ★ | ★ | ★ |
|  | Selection of the non-exposed cohort | ★ | ★ | ★ | ★ | ★ | ★ |
|  | Ascertainment of exposure | ★ | ★ | ★ | ★ | ★ | ★ |
|  | Outcome not present at start | ★ | ★ | / | ★ | ★ | ★ |
| Comparability | Comparability of controls | ★★ | ★★ | ★★ | ★★ | ★ | ★★ |
| Outcome | Assessment of outcome | / | ★ | ★ | ★ | ★ | ★ |
|  | Adequate follow-up duration | ★ | ★ | ★ | ★ | / | / |
|  | Loss to follow-up | ★ | ★ | ★ | ★ | / | ★ |
| Total score | | 8 | 8 | 6 | 9 | 9 | 9 |

*Note*: The study quality was assessed according to an adapted form of the Newcastle Ottawa Quality (NOS). This scale awards a maximum of 9 points to each study: 4 for selection, 2 for comparability, and 3 for assessment of outcomes. ★ = “one point”, ★★ = “two points”.

# Supplementary Table 6. Quality assessments of included cross-sectional studies

| **Items** | **Verier et al.**  **(2010) (21)** | **Jiang et al.**  **(2019) (26)** |
| --- | --- | --- |
| Define the source of information | ★ | ★ |
| List inclusion and exclusion criteria | ★ | / |
| Indicate time period used for identifying patients; | ★ | ★ |
| Indicate whether or not subjects were consecutive if not population-based; | ★ | ★ |
| Indicate whether there is an evaluator's subjective research component | ★ | ★ |
| Describe any assessments undertaken for quality assurance purposes | ★ | ★ |
| Explain any patient exclusions from analysis | ★ | ★ |
| Describe how confounding was assessed and/or controlled | ★ | ★ |
| Explain how missing data were handled in the analysis | / | / |
| Summarize patient response rates and completeness of data collection | ★ | ★ |
| Clarify what follow-up and the percentage of patients for which incomplete data | ★ | ★ |
| Total score | 10 | 9 |

*Note:* The study quality was assessed according to an adapted form of the Agency for Healthcare Research and Quality (AHRQ). This scale awards a maximum of 11 points to each study. ★ = “one point”.
